# Translatability of Animal Models for Alzheimer's Disease Using a Machine Learning Based Workflow

**DOI:** 10.1111/cts.70387

**Published:** 2025-11-11

**Authors:** Alex Foster‐Powell, Guy Meno‐Tetang, Amin Rostami‐Hodjegan, Kayode Ogungbenro, Donald E. Mager

**Affiliations:** ^1^ CAPKR, University of Manchester Manchester UK; ^2^ Neurocrine Biosciences London UK; ^3^ Certara Sheffield UK; ^4^ Division of Pharmacokinetics‐Pharmacodynamics and Systems Pharmacology, Department of Pharmaceutical Sciences University at Buffalo, SUNY Buffalo New York USA; ^5^ Enhanced Pharmacodynamics, LLC Buffalo New York USA

**Keywords:** 3×Tg, 5×FAD, Alzheimer's disease, APP/PS1, gene set enrichment analysis, machine learning, translational medicine

## Abstract

Despite significant investment, no effective disease‐modifying therapies for Alzheimer's disease (AD) have been developed to date. As understanding of the underlying causes of AD evolves, numerous animal models have been generated to study the disease. However, persistent therapeutic failures raise questions about the reasons for these shortcomings, including whether they stem from poor target selection and/or limitations in replicating key aspects of AD pathophysiology in animal models. In this study, a machine learning‐based workflow previously reported in the literature was modified and used to identify shared dysregulation in phenotype‐defining pathways across both animal models and human datasets—termed translatable pathways. This approach provided a framework for assessing the translational relevance of three widely used AD models: APP/PS1, 3×Tg, and 5×FAD, from hippocampal microarray data. The analysis suggested no translatable pathways in the APP/PS1 and 3×Tg preclinical models, whereas key pathways were identified in the 5×FAD (SREBP control of lipid synthesis and cytotoxic T‐lymphocyte pathways) model. Additionally, applying the workflow to publicly available microarray data from ibuprofen‐treated mice accurately predicted the clinical failure of ibuprofen for treating AD in human trials. This study highlights the importance of evaluating the translatability of animal models to human disease and provides a suitable framework for improving the selection of preclinical models in Alzheimer's research.

**Study Highlights**

- What is the current knowledge on the topic?
  ○ Current understanding of Alzheimer's disease (AD) underscores a significant translational gap between preclinical findings and human clinical outcomes. Numerous animal models, such as APP/PS1, 3×Tg, and 5×FAD, have been developed to mimic AD pathology, but consistent failures in clinical trials have raised concerns about their translational validity. Traditional differential gene expression methods often fall short in capturing conserved biological processes across species, leading to poor predictive utility in drug development.
- What question did this study address?
  ○ This study aimed to evaluate the translatability of these AD models by adapting a machine learning (ML)‐based workflow inspired by the TransPath‐C methodology. It employed sparse principal component analysis and support vector machines on pathway enrichment scores derived from microarray data, to identify conserved, phenotype‐defining biological pathways between human AD samples and mouse models.
- What does this study add to our knowledge?
  ○ The study found that the APP/PS1 and 3×Tg models lacked translatable pathways, while the 5×FAD model exhibited shared dysregulation in SREBP‐controlled lipid synthesis and cytotoxic T‐lymphocyte (CTL) activity—indicating higher relevance to human AD pathology. Importantly, this ML workflow predicted the clinical failure of ibuprofen in AD treatment based solely on mouse model data, demonstrating a degree of predictive validity.
- How might this change clinical pharmacology or translational science?
  ○ These findings advance our understanding by offering a robust computational approach to assess translational potential, shifting the focus from individual genes to conserved pathways. This could transform clinical pharmacology and translational science by enabling the preclinical identification of models and interventions more likely to succeed in humans. It may also lead to better‐informed decisions about which animal models to use for studying specific disease mechanisms and evaluating candidate therapies.

## 1 Introduction

Neurodegenerative diseases, such as Alzheimer's disease (AD), are chronic and multifactorial. It is currently estimated that between 15% and 20% of individuals over the age of 60 suffer from mild cognitive impairment [1]. As the population ages, this will lead to an increased care burden, with estimates projecting that there will be 100 million global cases by 2050 [2]. Since 1995, it is estimated that $42.5 billion of investment has been made in R&D in the private sector alone [3]. Despite such investment, only six novel drugs have been approved by the FDA, with only two disease‐modifying therapies (Lecanemab and Donanemab) and the controversial approval and subsequent withdrawal of Aducanumab, targeting the underlying pathology. As these compounds have only recently been approved, their effectiveness is not fully established and is only effective for early stage ad [ 4, 5, 6].

Treating AD presents significant challenges, as its underlying biology remains poorly understood. This lack of understanding complicates efforts to replicate the disease in preclinical models. Most animal models for AD, such as APP/PS1, 3×Tg, and 5×FAD, are transgenic and predominantly represent the familial form of the disease, which accounts for only approximately 5% of all AD cases [7]. In contrast, clinical trials are typically conducted in populations with sporadic AD, which constitutes the remaining 95% of cases. Consequently, although a treatment may appear promising in preclinical models and warrant progression to clinical trials, this success is often not replicated in human studies [8, 9]. The absence of positive outcomes in clinical trials raises concerns about the translational validity of the animal models currently in use. This string of failures has left the scientific community uncertain about the suitability of these models for developing effective treatments [10].

Traditionally, translatable biology between species or potential candidate drug targets may be identified through differentially expressed gene (DEG) analysis. Here, genes are individually scrutinized as to whether they are significantly over or under expressed in the diseased state compared to the control group [11]. This approach, however, has several limitations. Firstly, it evaluates only one‐to‐one homologs between the two species resulting in the loss of a significant amount of useful information. Secondly, individual gene behavior is often not well conserved across species [12]. Therefore, there is further information loss when only inspecting those genes that move in the same direction across the species. It is not surprising that only a few genes of interest are typically identified [13].

Computational approaches may help address these challenges, and innovative methodologies have been developed to assess the translatability of animal models [13, 14]. A principal component (PC) model using gene expression data (under diseased and healthy conditions) from a single species (typically mice) can be constructed to identify key features that account for variation in the data (often a result of organism phenotype). Human data may then be projected into this space to assess relative differences along mouse PCs [14]. Using these coordinates, a regression model (*TransComp‐R*) can assign phenotypes to data. This method has been applied to various diseases including inflammatory bowel disease, tuberculosis, and AD [14, 15, 16, 17]. It has also been extended into *TransPath‐C* [13], which introduces two fundamental innovations. First, it converts gene expression data into pathway enrichment scores, thereby preserving the integrity of organism‐specific data without the need for mouse‐to‐human homologs. This allows for the retention of valuable species‐specific information. Second, it utilizes a machine learning classifier to identify PCs that effectively distinguish data by phenotype.

In this study, a modified workflow, inspired by the *TransPath‐C* pipeline, was applied to microarray data from three widely used AD animal models (APP/PS1, 3×Tg, and 5×FAD) and used to assess their translatability to human datasets and identify phenotype‐defining pathways conserved across species. The approach was qualification tested through the prediction of clinical results using only preclinical microarray data and provided new insights into the relevance and translatability of these commonly utilized animal models of AD.

## 2 Methods

### 2.1 Data Selection and Quality Control

Microarray data from human postmortem brain tissue and three common AD mouse models (APP/PS1, 3×Tg, and 5×FAD) were extracted from publicly available datasets available through Gene Expression Omnibus (GEO). When selecting datasets, GEO was first queried from bulk human and mouse transcriptomic data. Data were selected with matching brain regions and microarray platforms and were further filtered using GEMMA [18], which is another database that can reanalyze gene expression data and assign a data quality score between 0 (low) and 1 (high) depending on the presence or absence of batch effect, data variability, and reproducibility. Only data with a GEMMA score ≥ 0.4 were selected. Ultimately, datasets were selected if they originated from the same brain region in both humans and mice and met all the inclusion criteria (Table 1).

**TABLE 1:** Overview of included datasets.

Brain region	Organism	Model	Number of diseased	Number of healthy	GEMMA score	GSE code
Hippocampus	Human	/	7	10	0.7	36,890
	Mouse	APP/PS1	5	4	0.6	67,306
		APP/PS1 (Ibu treated)	5	4		
		3×Tg	3	3	0.4	36,981
		5×FAD	3	3	0.9	50,521

### 2.2 Gene Set Enrichment Analysis (GSEA)

Raw data were background corrected and normalized via robust multichip averaging. If duplicate readings were available for a single gene, the repeat with the greatest value was selected. Independently for each dataset, the fold‐change expression for each gene was calculated as compared to the average of the control and ranked accordingly:

Next, pre‐ranked GSEA was performed in R for each sample using the package fgsea [19] to generate normalized pathway enrichment scores (NES), which allow for better comparisons between pathway enrichment scores regardless of the pathway length [20] and provide an indication of the extent to which a pathway is over or under expressed in a sample. BIOCARTA gene sets, used to convert gene expression into pathway enrichment scores, were accessed from the Broad Institute [20] for both humans (292 gene sets) and mice (252 gene sets). Gene sets containing more than 5 but less than 1000 genes and present in both human and mouse gene sets were selected for downstream analysis. This resulted in a total of 237 pathways and a pathway enrichment matrix of p pathways × n samples for each dataset.

### 2.3 Sparse Principal Component Analysis Model Development

**FIGURE 1:**
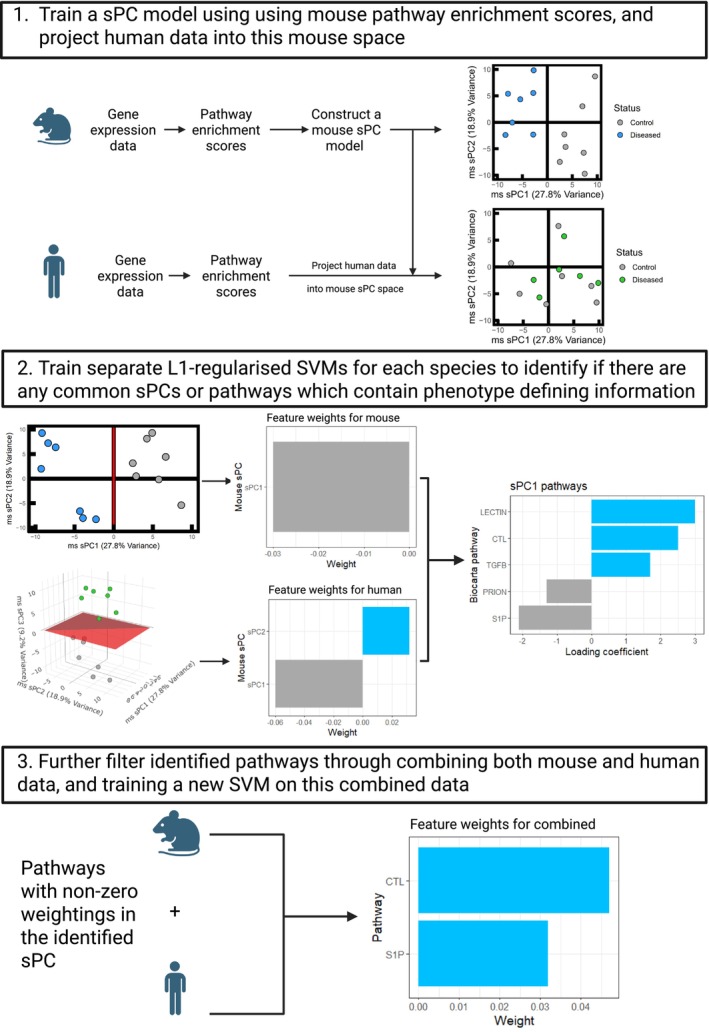
Overview of the TransPath‐C inspired workflow. Key steps include, conversion of gene expression data to pathway enrichment scores, construction of a mouse sPC model into which human data is projected, and the development of species‐specific SVMs.

For each dataset, it was assumed that a large proportion of the variance in the data is a result of the species' different phenotypes. To determine which pathways are responsible for this variation in the data, and to reduce signals associated with noise, a sparse principal component (sPC) model may be developed [21] (Figure 1). For this, the NES from the mouse and human datasets were individually power transformed (Yeo‐Johnson [22]) to have a mean of zero and a variance of one for each pathway across each species' samples using the PowerTransformer package in Python [23]. Next sPC models were generated for each mouse dataset using the sparsepca package in R following penalty parameter optimisation [24]. Mouse datasets were selected to build the sPC models, owing to the homogeneous nature of mouse models, and thus the clearer expected clustering of the data by phenotype. Here, a regularization penalty term, *α*, was introduced to penalize the loadings in the principal components, which was varied between 10^−6^ and 0 over 10 divisions. For each *α* term tested, leave‐one‐out cross‐validation (LOOCV) was used to determine the mean and variance of the cumulative variance captured by the first three sPCs. The optimum *α* was the largest penalty term (indicating a sparse model) that still captured ≥ 50% of the total variance within a dataset in the first three sPCs. The final sPC model was built using mouse data and the optimum *α* term. The human pathway enrichment matrix from the same brain region was then projected into each mouse sPC model through matrix multiplication by the eigenvectors of the developed mouse models. The proportion of variance explained by each mouse sPC in both the human and mouse data was then calculated.

### 2.4 L1‐Regularized Support Vector Machine (SVM) Learning

For each mouse sPC model, the individual projections of the mouse and human samples in the mouse space were labeled according to their phenotype (0: healthy, 1: diseased). For each species and mouse model system (APP/PS1, 3×Tg or 5×FAD), distinct L1 regularized support vector machine (SVM) models were built using the LinearSVC function in the scikit‐learn package in Python [23]. Using LOOCV and balanced class weights, the optimal L1 penalty hyperparameter, *C*, was determined by the maximization of the F1 score. A parameter grid ranging from 10^−3^ to 10^2^ was explored using the GridSearch function. Using this optimized *C* value, the entire dataset was used to train the final SVM classifier for each model system. The final model performance was evaluated through inspection of the F1 score, model accuracy, and area under the receiver operating characteristic curve (AUC ROC). The final SVM model was utilized to reduce the dimensionality of the sPCs necessary for accurate classification of the data. Only those sPCs with nonzero loadings (indicating their use by the SVM to classify the data) were retained for subsequent analysis.

### 2.5 Identification of Translatable Pathways

sPCs with nonzero weights utilized by the SVMs to classify the mouse and human data were recorded. If the SVM models for both mouse and human data shared a common sPC, it may suggest that this sPC contained phenotype‐defining information that is conserved across both species. However, owing to the nature of sPCs, multiple pathways were grouped together depending upon how much variance in the mouse data they captured. Although useful for initially filtering phenotype‐relevant pathways, not all pathways may be required to be analyzed to assign a phenotype. Therefore, an additional filtering step was performed on any initially overlapping pathways to produce a final list of translatable pathways.

For model systems in which common sPCs were identified by the first SVM, the relevant mouse and human NES scores were combined into a single new dataset. This combined dataset was then power transformed, and a new SVM model was trained on the new dataset for classifying disease status. The model was not informed of the species from which the data had originated. Finally, any nonzero weighted pathways (i.e., translatable pathways) were identified, which are associated with the phenotype irrespective of the species.

### 2.6 Qualification Test With Preclinical Drug Data

The GSE67306 dataset also contained microarray data from the hippocampus of APP/PS1 mice following 1 month of chow spiked ibuprofen (Ibu) treatment. As a step toward method qualification testing, the phenotype of the new Ibu test data was evaluated using overlapping sPCs identified by the SVM model trained using untreated APP/PS1 and control mice. If no sPCs were identified in the original SVM, *α* was reduced until overlapping sPCs were identified. For this Ibu dataset, gene expression data were converted to pathway enrichment scores (as described above). These data were then power transformed using the same transformation parameter identified in the untreated APP/PS1 datasets, using the transformation function in the PowerTransformer package in Python, and projected into the mouse sPC model. The untreated APP/PS1 SVM developed using the reduced *α* value was finally used to classify treated subjects in the Ibu dataset as either diseased or healthy.

## 3 Results

### 3.1 sPC Model Development

**FIGURE 2:**
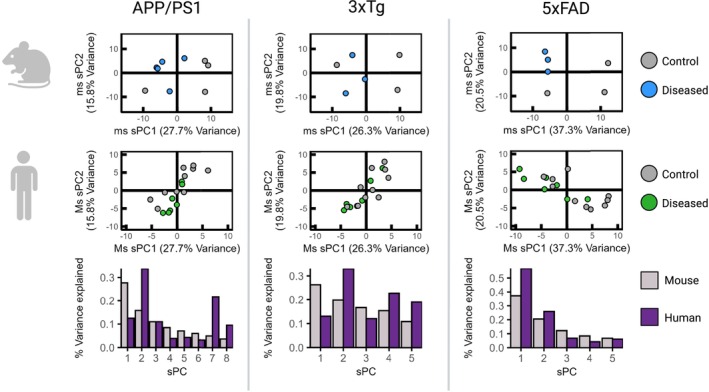
Development of sPC models using pathway enrichment scores for each mouse model (row 1), and the projection of the same human pathway enrichment scores into this model through eigenvector multiplication (row 2). The variance captured by each sPC for both the mouse and the human data is shown in row 3.

Following optimisation, an *α* of 10^−2^ was selected for all datasets, as this retained > 50% of the variance of the mouse data for all models. The developed sPC models generally divided the mouse data well, with the 5×FAD data showing the clearest separation (Figure 2). Following projection of human data into each mouse sPC model, no clear separation was observed along sPC 1 and 2. For human data projected into the mouse APP/PS1 sPC model, the greatest proportion of variance was captured by mouse sPC 2 and 7 (33%, 22%). For human data projected into the mouse 3×Tg sPC model, the greatest proportion of variance was captured by mouse sPC 2 and 4 (33%, 23%). sPC 1 and 2 accounted for the most variance in the human data projected into the 5×FAD mouse sPC model (57%, 26%), with each subsequent sPC explaining a reduced proportion of the variance in the human data.

### 3.2 SVM Classifier

**FIGURE 3:**
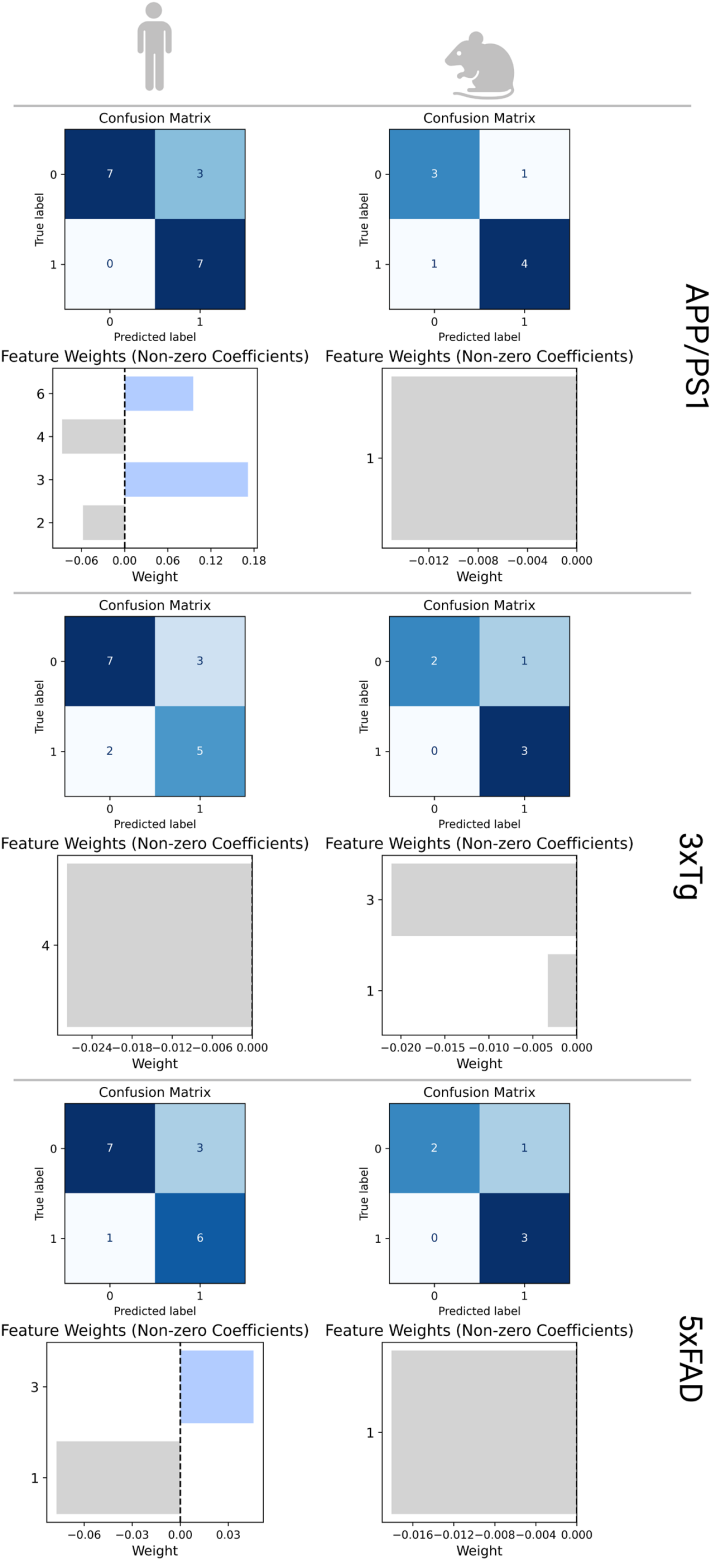
Confusion matrices for the L1‐regularized SVM classifiers using power transformed human and mouse pathway enrichment scores, for each animal model. The corresponding nonzero sPCs utilized by each L1‐regularized SVM and their weights for both species are presented below the confusion matrix for each SVM model.

**FIGURE 4:**
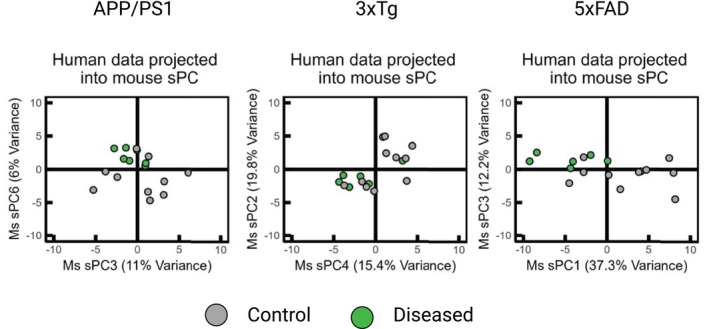
Confirmation of the identified mouse sPCs required by the SVM to classify the phenotype of human data. If only one sPC was identified by the SVM (3×Tg), the next sPC which accounted for the most variance in the human data was used for visualization. Plotting of the mouse sPCs identified by the SVM to classify the human data demonstrates much clearer separation of the human data, as opposed to inspecting sPC1 and sPC2 as in the mouse data.

SVM classifier models were developed independently on the sPC loadings for each mouse model and human data projected into the mouse sPC space (Figure 3). For each model, a regularization hyperparameter, *C*, was optimized using LOOCV to maximize the F1 score. For mouse and human SVMs respectively, *C* values of 0.019, 0.159 (APP/PS1), 0.17, 0.022 (3×Tg) and 0.018, 0.058 (5×FAD) were selected. The SVM models demonstrated good accuracy for both human and mouse data across different models: APP/PS1 (0.78, 0.82), 3×Tg (0.83, 0.71), and 5×FAD (0.83, 0.75). This performance was achieved by evaluating specific sPCs in each model (Figure 3). For the APP/PS1 model, accuracy was assessed using sPC1 in mouse data and sPCs 2, 3, 4, and 6 in human data. In the 3×Tg model, sPC1 and sPC3 were used for mouse data, whereas sPC4 was used for human data. For the 5×FAD model, performance was based on sPC1 in mouse data and sPCs 1 and 3 in human data. Plotting of these identified human sPCs, greatly improved the clustering of the human data in the mouse sPC models (Figure 4).

### 3.3 Mouse Phenotype Defining Pathways

The phenotype of the APP/PS1 mouse model was characterized by variance in pathways associated with immune response (33%), cell survival (31%), neuronal health (21%), and protein misfolding (8%), with the remainder involving oxidative stress response and metabolic regulation (Table S1). For the 3×Tg model, variance was primarily driven by immune response (38%), cell survival and cell death regulation (22%), neuronal health (13%), and cell cycle regulators (13%). Additional pathways were linked to misfolded protein sorting, stress responses, dysfunctional nucleocytoplasmic trafficking, and DNA repair. The 5×FAD model exhibited variance across 26 pathways, with a predominant focus on immune response (53%) and neuronal health (13%). Pathways related to protein aggregation (6%) and cell cycle regulation (9%) were also notable, and the remaining pathways were associated with oxidative stress response and chromatin remodeling.

### 3.4 Identification of Translatable Pathways

No overlapping sPCs were found to separate human and mouse data in either the APP/PS1 or 3×Tg models (Figure 3). Furthermore, no pathways with nonzero loadings were identified in the selected sPCs. However, sPC1 was used by both the mouse and human SVM to classify the data in the 5×FAD dataset (Figure 3). This corresponded to 26 pathways, which contained phenotype‐defining information in both human and mouse data.

**FIGURE 5:**
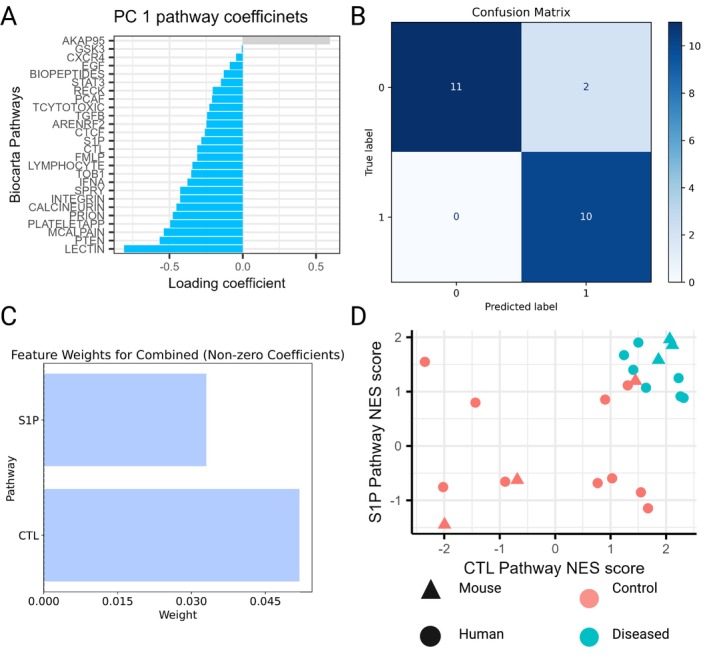
sPC1 was identified by the SVMs to predict the phenotype of the human and 5×FAD mouse dataset. This corresponded to 26 pathways (A). A second SVM was then trained using just these 26 pathways to classify a combined pool of human and mouse data and the performance was assessed with a confusion matrix (B). This model performance was achieved through inspection of the S1P and CTL pathways, identifying these as phenotype‐defining in both mice and humans (C). (D) Separation of data, irrespective of species through inspection of only the S1P and CTL pathways.

Owing to the grouping nature of sPCs, it was evaluated whether all these pathways were required in order to predict an organism phenotype. NES scores of the 26 identified pathways from both the mouse and human data were selected, combined into a new single dataset, and power transformed. A new L1‐regularized SVM model was trained on this combined dataset, and the combined SVM model performed well for accuracy (0.91), ROC AUC (1.0) and F1 score (0.91). This model performance was achieved with a *C* of 0.033 and evaluating power transformed NES scores for SREBP induced lipid synthesis (S1P) and cytotoxic T‐lymphocyte (CTL) pathways only. A plot of normalized enrichment scores (NES) for the two identified pathways, S1P and CTL, revealed a distinct separation between disease and control clusters independent of species (Figure 5). Control samples exhibited a broader range of NES values, whereas diseased samples consistently displayed elevated NES scores for both pathways. In the mouse data, a strong correlation between these pathways was observed, which was not evident in the human data. This suggests that both S1P and CTL NES scores need to be considered for classifying human data.

### 3.5 ML Workflow Confirms Ibuprofen Should Not Impact Disease Progression

**FIGURE 6:**
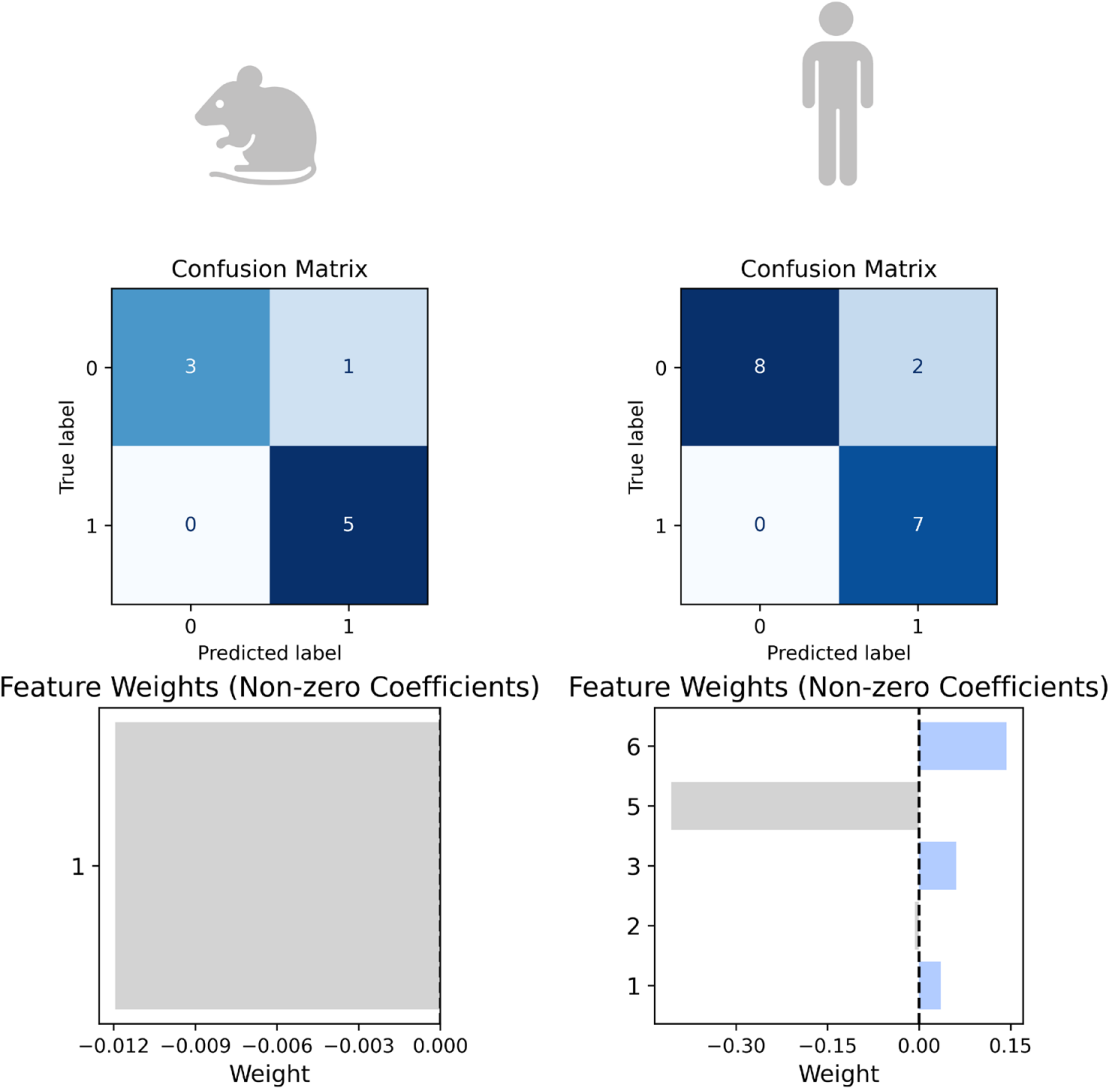
SVM construction to qualify test the computational workflow, with the introduction of Ibu‐treated APP/PS1 mouse data. New SVMs were trained on mouse and human coordinates in the APP/PS1 untreated sPC model, following the reduction of *α* in the sPC construction. This identified sPC1 as a common sPC between both species. Model performance was assessed through confusion matrices, indicating model accuracy and f1 score, in addition to AUC ROC.

For qualification purposes, the final workflow was used to test whether identified pathways could be used to anticipate a drug failure for treating AD in humans. Unfortunately, it is not common practice to publish array data from brain tissue following drug treatment in mice. However, a publicly available dataset from APP/PS1 mice treated with ibuprofen was available in the literature. Initially, using the predefined threshold for *α* value selection, the APP/PS1 mouse data did not identify any translatable pathways. Therefore the *α* penalty term in the sPC model construction was decreased to 4 × 10^−4^. From this lower inclusion criteria, sPC1 was identified by the SVM in both the mouse and human (Figure 6). The SVMs for both mouse and human classification performed well with a lower *α* for ROC AUC (0.75, 0.96), F1 score (0.90, 0.82), and accuracy (0.89, 0.82). The phenotype of the power transformed ibuprofen treated APP/PS1 data was predicted for humans using this trained SVM model, and 80% (4/5) of samples were classified as diseased and 75% (3/4) of control samples were assigned as healthy.

## 4 Discussion

To overcome the current stagnation in the development of disease‐modifying therapies for AD, the identification of robust, translatable experimental models is essential. A ML‐based platform has been developed to evaluate the translational relevance of animal models, offering a means to identify features that are phenotype defining in both animals and humans [13]. In this study, a workflow inspired by the *TransPath‐C* framework was utilized to assess three commonly used AD mouse models. Broadly elevated immune responses were found across diseased animal models, but poor translatability was suggested for the APP/PS1 and 3×Tg models. In contrast, the 5×FAD mouse model demonstrated stronger alignment with human data, highlighting two cross‐species phenotype defining pathways—SREBP control and lipid synthesis and CTL.

The translatable validity of this approach has been documented previously in the literature [14, 16]. In IBD, the precursor to *TransPath‐C*, or *TransComp‐R*, which uses the same principles, identified elevated ITGA1 expression as a driver of anti‐TNF therapy resistance in patients: a molecular feature discernible in the mouse data that was predictive of patient phenotype. This finding was confirmed with single‐cell RNA sequencing of human biopsies and functionally validated using patient‐derived immune cells [14]. Similarly, when applied to tuberculosis (TB) data [16], the infection‐induced unfolded protein immune response was found to be a signature in mouse data and was highly predictive of human TB status. Inhibition of this pathway in infected mouse bone‐marrow‐derived macrophages led to a reduction in the inflammatory response. Together, these results demonstrate the utility of this workflow in identifying phenotype‐defining features that are conserved across species.

Microarray data from the hippocampus was selected for this analysis. AD is believed to originate from the hippocampus, making this region of high priority and where data is most abundant. As the origination point, it is most commonly impacted in the diseased population, and thus assumed to show clearer separation between diseased and healthy groups in the analysis. It would be of interest to analyze other brain regions impacted through disease progression (e.g., cortex) to evaluate if findings hold. However, this would likely require a larger population to produce meaningful results, due to the variable impact across brain regions in the heterogeneous disease population.

As a step toward qualification, the workflow was applied to Ibu‐treated APP/PS1 mice, and using only overlapping sPCs, the SVM was used to classify samples. Ibu‐treated mice were predominantly classified as being still in a diseased state, contrary to the conclusions reached when observing only mouse behavior [25]. This lack of efficacy has been confirmed in multiple, large‐scale clinical trials, in which no significant difference in disease status was identified in humans [26, 27]. The ability of the workflow to anticipate this lack of efficacy is encouraging; however, a reduced *α* threshold was needed, which introduces more noise and increases the risk of overfitting. These results are encouraging and provide qualification, but larger and more diverse datasets will be necessary to strengthen its robustness and generalizability.

Examination of the PCs identified by the SVMs to classify the mouse phenotypes in the three animal models confirmed that there were a greater absolute number and relative proportion of inflammatory pathways in the 5×FAD mouse model compared to the 3×Tg and the APP/PS1 models. This aligns with literature reports suggesting an increased degree of neuroinflammation in the 5×FAD over APP/PS1 [28]. Dysregulation of the immune response was also identified as a translatable signature in TASTPM mice (an alternative AD model) following analysis using the *TransComp‐R* approach [15]. Together, this indicates that neuroinflammation is a common phenotype defining feature in all these animal models. However, whether this is a result of the disease or a driver behind disease progression is yet to be determined.

No translatable pathways were identified between the mouse and human hippocampus datasets in the APP/PS1 and 3×Tg models. Projection of the human data into the mouse sPC models revealed differences between the two systems, as reflected through the proportion of variance captured by each sPC (Figure 2). Almost all pathways in the PCs identified by the SVM for classification of the human data had strong literature support for their dysregulation in AD patients (Table S1). This suggests that one‐to‐one mapping between mouse and human datasets is not feasible, likely owing to fundamental differences in the underlying biological drivers of variation [16]. This also indicates that key biological signals may be over‐ or under‐ represented in the mouse model, underscoring the necessity for computational approaches to address such translational challenges.

Analysis of the 5×FAD mouse model identified two translatable pathways—SREBP control of lipid synthesis (S1P) and CTL—consistently upregulated across species (Figure 5D). The SREBP‐2 pathway is particularly compelling, given its association with AD risk [29] and tau pathology [30]. Disruption of lipid metabolism by causal AD genes in 5×FAD mice [31] further implicates SREBP‐2 in disease mechanisms. Upregulation of SREBP‐2 in 5×FAD‐DHCR24 knock‐in mice reversed cognitive impairment and reduced hallmark AD pathologies, reinforcing its therapeutic relevance. Given the broad influence of lipid signaling on cellular responses, its disruption may underlie the widespread dysregulation observed across multiple pathways [32]. As interest in SREBP‐2 targeting grows [33], these findings provide compelling preclinical evidence for its potential in disease modification, suggesting that similar therapeutic benefits may extend to humans.

The second translatable pathway identified was associated with cytotoxic T‐cells, which similarly have been implicated in the pathology of AD (albeit with a slightly more pleiotropic function) [34, 35, 36, 37, 38]. In mice, the S1P and CTL pathways seemed highly correlated, suggesting a potential mechanistic relationship between lipid synthesis and CTL activity. A possible driver for this relationship is the role that S1P signaling plays in CD8+ cell response [39, 40]. This correlation was not present in the human data, possibly because of alternative pathways involved in CTL activation in humans or potentially owing to the heterogeneity of human disease. Subsequently, in humans, the phenotype could not be assigned by inspection of a single pathway, and instead relied on a combination of pathways. This fits with the emerging consensus that a single marker is not necessarily appropriate to assign phenotype [41]. Together, these findings suggest that the 5×FAD mouse model could be suitable as a preclinical model for CD8+ T‐cell activation (possibly through S1P signaling).

The limitations of the current study are primarily owing to the availability of data, especially following drug treatment. Analysis was performed with hippocampus tissue in late‐stage AD, using one microarray platform on a relatively small dataset. Therefore, repeating this analysis on a larger dataset has the potential to identify different translatable features. Confidence in the pathways identified will come as further analysis is performed on larger datasets, or data generated through different array platforms. Subsequent analysis may reveal the repeat occurrence of the same specific or class of pathway (e.g., inflammatory, lipid signaling) and further provide confidence in the wider translatability of the animal model and confidence in this approach. Despite the small sample size, final identified pathways were in agreement with the literature. Another limitation is that information on comorbidities or concomitant medications was not available, which precluded a detailed investigation into further sources of patient heterogeneity. As larger, high‐quality datasets from brain regions of interest become available for both humans and mice, it will be possible to explore these aspects of heterogeneity more thoroughly.

The proposed workflow was developed for use with array data; however, this approach is also compatible with data from other sources and applicable to various diseases. Integrating RNAseq data, biomarker measurements (blood or CSF‐based), and/or PET scans could customize the results generated by this pipeline to address specific research questions, such as identifying early‐stage disease markers. Furthermore, the identified system features could enhance the utility of PK/PD or QSP models, reducing the reliance on Aβ burden or behavioral alterations in less relevant animal models as a proxy for disease status.

## Author Contributions

A.F.‐P. and D.E.M. wrote the manuscript, K.O., A.R.‐H., D.E.M., and G.M.‐T. designed the research, A.F.‐P. analyzed the data, A.F.‐P. performed the research.

## Conflicts of Interest

The authors were affiliated with the following institutions at the time of writing: Alex Foster‐Powell (University of Manchester), Guy Meno‐Tetang (AstraZeneca Neuroscience, United Kingdom), Amin Rostami‐Hodjegan (University of Manchester and Certara), Donald E. Mager (University at Buffalo, SUNY and Enhanced Pharmacodynamics LLC), Kayode Ogungbenro (University of Manchester). All other authors declared no other conflicts of interest.

## Supporting information


**Data S1:** Power transform.

**Table S1:** Pathways identified by the SVM for the APP/PS1, 3×Tg and 5×FAD mouse, and human data. Where present, references are provided which implicate the pathways in AD.

**Data S2:** SVM_classifier.

**Data S3:** Full model code (R).
